# Current status and progress of laparoscopic inguinal hernia repair: A review

**DOI:** 10.1097/MD.0000000000034554

**Published:** 2023-08-04

**Authors:** Li-shuai Xu, Qian Li, Ye Wang, Jia-wei Wang, Song Wang, Cheng-wei Wu, Ting-ting Cao, Ya-bin Xia, Xiao-xu Huang, Li Xu

**Affiliations:** a Department of Gastrointestinal Surgery, The First Affiliated Yijishan Hospital of Wannan Medical College, Wuhu, China; b Key Laboratory of Non-coding RNA Transformation Research of Anhui Higher Education Institution, Wannan Medical College, Wuhu, China.

**Keywords:** inguinal hernia, LIHR, TAPP, TEP

## Abstract

After 30 years of development, laparoscopic inguinal hernia repair (LIHR) has become the main method for treating adult inguinal hernia. LIHR is more standardized, the approach of single-port laparoscopic hernioplasty, the advantages of robotic inguinal hernioplasty, the application of new patches and the selection of surgical methods for different populations have become the focus and difficulty of current research. This article summarized the research progress of LIHR in recent years. Different keywords and phrases including inguinal hernia, LIHR, transabdominal laparoscopic preperitoneal hernia repair, and total extraperitoneal hernia repair were used to search the PubMed, China National Knowledge Infrastructure, and Web of Science databases for related original and review articles that serve the aim of this article well, which was to perform a nonsystematic review of the development, progress, and current status of LIHR.

## 1. Introduction

Inguinal hernia is a common clinical condition, and it is estimated that more than 20 million inguinal hernia surgeries are performed worldwide each year.^[1]^ The occurrence and development of inguinal hernia are closely related to congenital factors, such as patent processus vaginalis and dysplasia of the groin, acquired factors, such as anatomical abnormalities caused by advanced age, stunted growth, and malnutrition, and other factors, such as abdominal wall muscle weakness or increased abdominal pressure, which can in turn be caused by multiple factors.^[2,3]^ The prevalence of inguinal hernia increases with age.^[4,5]^ According to data from 2017, the cumulative prevalence of inguinal hernia in men aged 25 to 34 years was 5%; 35 to 44 years, 10%; 45 to 54 years, 18%; 55 to 64 years, 24%; 65 to 74 years, 31%; and 75 years and above, 45%. The incidence of inguinal hernia is 8 times higher in men than in women, and 90% of patients undergo inguinal hernia repair.^[6]^ Currently, inguinal hernia in adults can only be cured by surgical treatment methods. Surgical approaches have evolved from different open tissue repair techniques to the use of patches and eventually the widespread use of minimally invasive techniques. Laparoscopic inguinal hernia repair (LIHR) has evolved over the past 30 years and has reached a basic level of maturity in terms of procedure and technique. LIHR has the advantages of minimal invasiveness, favorable aesthetics, mild postoperative pain,^[7]^ and fast recovery. The recurrence rate is not significantly different from that of traditional tension-free repair, and the incidence of postoperative complications is lower than that of traditional tension-free repair.^[8]^ This manuscript summarizes the development, progress and current status of LIHR.

## 2. History of laparoscopic inguinal hernia repair (LIHR)

In the context of the rapid development of modern medicine, various surgical modalities for the treatment of inguinal hernia through laparoscopic techniques have arisen. In 1982, Professor Ralph Ger first proposed the theory of LIHR, which became popular in late 1990 and was gradually phased out due to the lack of abdominal wall defect repair and the high rate of postoperative recurrence.^[9]^ In 1991, Fitzgibbons reported the direct use of mesh to cover and repair the hernia ring opening and surrounding tissues via the abdominal cavity. This method, called intraperitoneal onlay mesh (IPOM) repair, yields good early results, but carries the risk of serious postoperative complications, such as intestinal obstruction, intestinal perforation and abdominal erosion; at the same time, the mesh patch is prone to displacement, leading to a high hernia recurrence rate.^[10]^ In 1990, Schultz reported a method consisting of hernial sac tamponade with mesh repair, which still shows a recurrence rate of 25%. However, this method mainly involves cutting the peritoneum and placing the patch directly in front of the peritoneum, which reduces the occurrence of abdominal intestinal adhesion. This method was improved through continuous research with biomechanical and anatomical analysis, leading to increases in the area of the hernia mesh, removal of the hernia mesh filling, and eventually the development of transabdominal laparoscopic preperitoneal (TAPP) hernia repair.^[11,12]^ McKernan was the first to report a completely extraperitoneal laparoscopic repair method, called total extraperitoneal (TEP) hernia repair.^[13]^

## 3. Current research status of various surgical methods for LIHR

### 3.1. IPOM repair: a pioneer LIHR method

IPOM repair is the earliest reported LIHR method. Because the patch is placed over the hernial defect and fixed at that location under laparoscopy without peritoneal separation, the postoperative pain is obvious, the patch may easily shift, and the recurrence rate is high.^[14,15]^ To date, all major guidelines and hospitals around the world have clearly noted that IPOM repair is not recommended as a treatment for primary inguinal hernias.^[16]^ The IPOM procedure is now mainly indicated for the treatment of incisional hernias. Among those with inguinal hernias, IPOM repair is limited to those who have experienced recurrence many times, especially those who have undergone patch implantation in the preperitoneal space.^[17–19]^ The technical aspects of IPOM repair have been improved, resulting in methods such as transabdominal partial preperitoneal repair.^[20–22]^ The design concept is perfect, but the execution is limited by technical bottlenecks related to materials.^[23]^

### 3.2. TAPP and TEP repair: currently widely used LIHR methods

**Figure 1.**
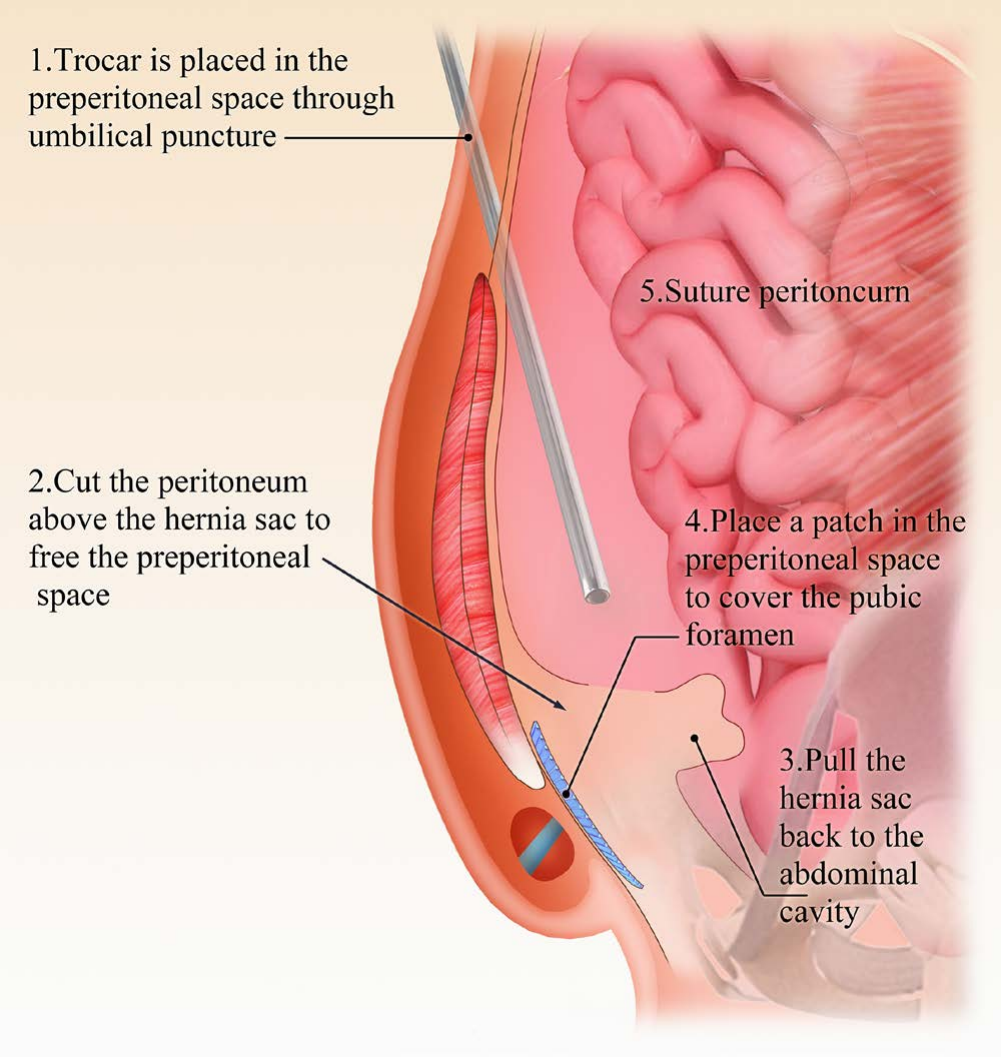
TAPP surgical procedure diagram. TAPP = transabdominal laparoscopic preperitoneal hernia repair.

The TAPP procedure is still the most commonly performed LIHR procedure.^[24]^ The operation steps generally include the following: A supraumbilical or subumbilical viewing hole into the abdominal cavity is created to explore the abdominal cavity and inguinal region and determine the type and stage of the hernia. Approximately 4 cm above the hernia ring, the peritoneum is incised in an arc from the medial umbilical ligament to the anterior superior iliac spine, and the medial pubic bladder gap (Retzius gap) and the lateral iliac fossa gap (Bogros’ gap) are incised, allowing lateral exposure to the iliopubic bundle, anterosuperior exposure up to the iliac spine, medial exposure exceeding the pubic symphysis, and inferomedial exposure exceeding the pectineal ligament by 2 cm. The hernial sac, spermatic cord, and vas deferens are identified and mobilized, and the hernial sac is transected or completely dissected. A patch is positioned to cover the entire area of weakness in the anteroinferior abdominal wall and fixed in place.^[25]^ Finally, the peritoneum is closed. The TAPP procedure is performed directly in the abdominal cavity, with a wide surgical field allowing easy identification of anatomical structures (Fig. 1).

**Figure 2.**
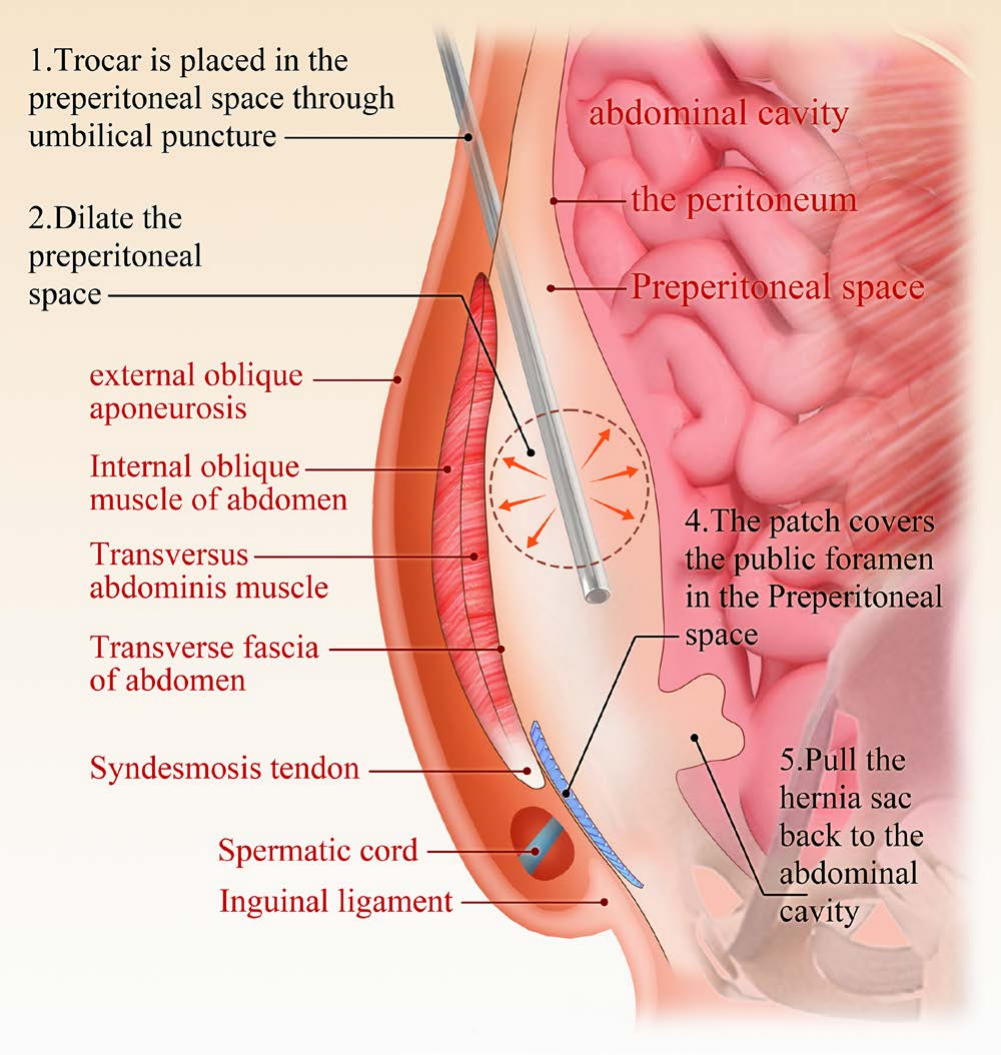
TEP surgical procedure diagram. TEP = totally extraperitoneal hernia repair.

The TEP procedure does not require entry into the abdominal cavity and instead only requires the creation of a space between the abdominal wall and the peritoneum, making it easier to reach the location of the abdominal wall defect and repair it with a patch. In this procedure, after a 1.0-cm-long subumbilical incision is made and the skin, subcutaneous connective tissue and anterior rectus abdominis sheath are incised, the posterior rectus abdominis sheath is exposed, mildly dilated and directly separated to create a gap between the dorsal rectus abdominis muscle and the posterior sheath, which is widened by blunt separation. The anterior peritoneal space can be accessed by the balloon method, mirror push method, retropulsion method or finger separation method.^[26]^ Then, the laparoscope is placed, pneumoperitoneum is established, and structures in the inguinal region, such as the closed artery, foramen and spermatic cord, are dissected extraperitoneally; subsequently, the hernial sac is moved into the peritoneal cavity, and the patch is placed behind the muscle.^[27,28]^ Successful establishment of the extraperitoneal cavity is essential to ensure the success of TEP repair. Especially when entering the Bogros hiatus from the Retzius hiatus, hernial sac dissection may easily lead to peritoneal injury. When the peritoneum is damaged, gas enters the peritoneal cavity and elevates the peritoneum, resulting in narrowing of the surgical space and compromising the surgical view. If the peritoneal injury rupture is small, it can be sutured or closed with absorbable clips first; if the rupture is large, seriously affects the surgical operation and is difficult to suture, or if the patient has a history of previous abdominal surgery resulting in localized adhesions in the anterior peritoneal space to the abdominal wall that prevent the dissection, the procedure should be converted to TAPP repair (Fig. 2).

There are several similarities and differences between TAPP and TEP repair. While the LIHR procedure has evolved since its introduction in the early 1990s, from TAPP to TEP repair, and the plane of separation and patch placement is the same, i.e., preperitoneal, in both approaches. In patients, significantly less time is required for TEP than TAPP repair, possibly because TAPP repair requires a peritoneal incision and final suturing, which increases the number of steps and prolongs the operation. However, TEP repair is superior to TAPP repair in terms of postoperative pain because of the relatively short duration of the TEP procedure, the lack of access to the peritoneal cavity and the absence of peritoneal sutures.^[29]^ Both methods of patching require complete coverage of the entire extent of the pubococcygeal muscle foramen. However, these procedures also have unique characteristics. TAPP repair requires access to the peritoneal cavity to open and close the peritoneum, which offers a large surgical field and allows easy identification of anatomical structures. The surgical technique is relatively simple, but the abdominal organs are easily affected during the procedure. In contrast, TEP repair allows complete separation of the anterior peritoneal space through the extraperitoneal cavity without entering the abdominal cavity, which has little impact on the abdominal organs. Technically, TEP repair is more reasonable, as it is accomplished without entering the abdomen, but the surgical field is small, and it can be relatively difficult to identify anatomical structures. The peritoneum can be easily damaged if the procedure is not completed correctly. In addition, the surgical field becomes narrower after the gas enters the abdominal cavity, which increases the difficulty of the operation and prolongs the learning curve. Nevertheless, there was no significant difference in the length of postoperative hospital stay, complication rate, or recurrence rate between patients treated with the 2 procedures.^[30,31]^

**Figure 3.**
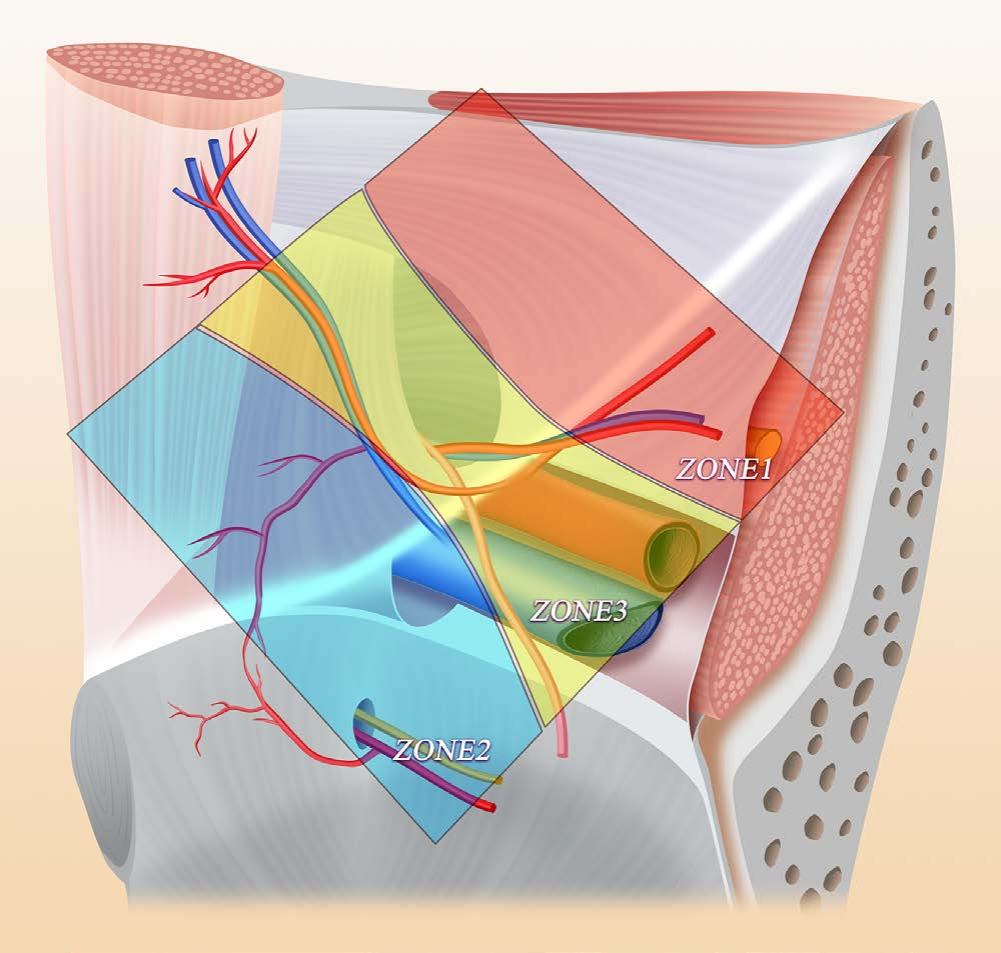
Three zones of preperitoneal space dissection.

**Figure 4.**
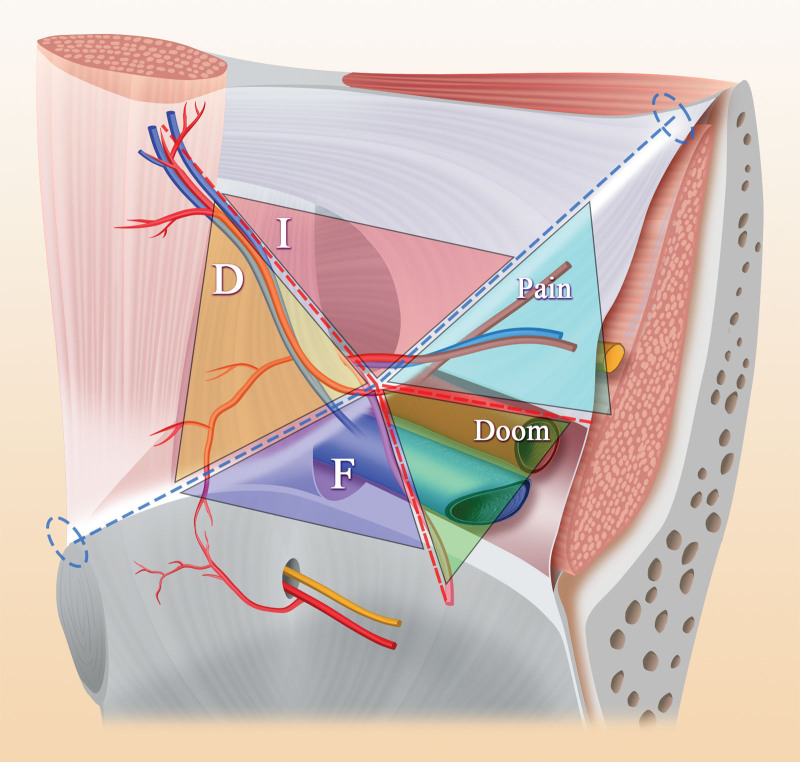
The inferior abdominal wall vessels, vas deferens and spermatic cord vessels form an inverted Y shape, and the iliopubic bundle passes through the inverted Y to form a schematic diagram of 5 triangles, the 5 areas are respectively: oblique hernia (I), pain, doom, femoral hernia (F) and direct hernia (D).

Some scholars have proposed the concept of the inverted Y and 5 triangles regarding the anatomy in the inguinal region.^[32]^ According to this new anatomical concept, the inguinal region is subdivided into 3 zones: zone 1, corresponding to the lateral region of the deep inguinal ring and spermatic cord blood vessels; zone 2, including the inferior abdominal blood vessel and the medial side of the vas deferens and corresponding to direct hernias; and zone 3, including the inferior abdominal wall vessels, the deep inguinal ring, the spermatic cord unit and the external iliac vessels, which are surgical areas that need more attention (Fig. 3). On this basis, 10 guidelines have been proposed: 1: In TAPP repair, the peritoneal incision should be at least 4 cm above the edge of the deep inguinal ring, and the open flap should extend from the anterosuperior iliac spine to the medial umbilical fold. 2: Dissection should strictly follow the peritoneal plane; in TAPP, dissection should start in zone 1 or 2 and end in zone 3, while in TEP repair, dissection is usually performed in zone 2 first, followed by zones 1 and 3. 3: The separation should extend at least 2 cm below the pubic symphysis and pubic area to create sufficient space to accommodate a mesh patch of an appropriate size, which should overlap the direct triangle and femoral triangle by at least 3 to 4 cm; notably, removing normal fat plugs from the obturator canal is unnecessary and may cause bleeding and is therefore not recommended. 4: The external iliac vein can be visualized to avoid the omission of femoral hernias in zone 3. 5: It is sufficient to dissect the peritoneum downward until the vas deferens passes through the level of the external iliac vein in zone 3 and the iliopsoas muscle behind zone 1. 6: For large hernias or inguinal scrotal hernias, it is recommended to transect and discard the distal hernial sac in the scrotum. 7: The deep inguinal canal should be explored when dissecting the third area to find any spinal lipomas. 8: A large patch should be placed to cover the pubic foramen, with at least 3 to 4 cm of overlap. 9: Fixation of the patch is unnecessary, especially in TEP repair. 10: Deflation should be performed under direct visualization^[33,34]^ (Fig. 4).

At present, the common views on the selection of TAPP or TEP repair are as follows: TAPP repair is preferred for patients with a long onset history, large hernial sac, incarcerated hernia, recurrent hernia, and history of sclerotherapy injection and for female patients.^[35–39]^ Patients with a history of abdominal surgery need to be selected based on the incision location and surgical site. In general, the TEP method is preferred in cases requiring lateral abdominal surgery (e.g., appendectomy), upper abdominal surgery, and surgier with a transverse lower abdominal incision or a paramedian incision, while the TAPP method is preferred in cases requiring a median lower abdominal incision and in patients with a history of bladder surgery.^[40–42]^ TEP repair is preferred for straight hernias, bilateral hernias, and hernias with a short history.^[43,44]^ TEP repair is preferred in elderly patients when they are suitable for both TEP and TAPP repair.^[45,46]^

### 3.3. Single-incision laparoscopic surgery (SILS): an innovative LIHR method

**Figure 5.**
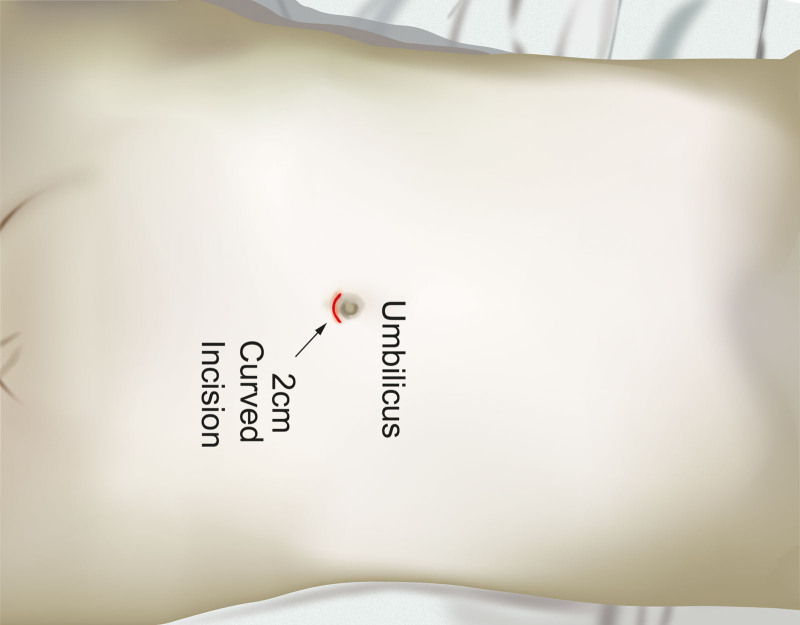
Design of incision for posterior sheath approach.

**Figure 6.**
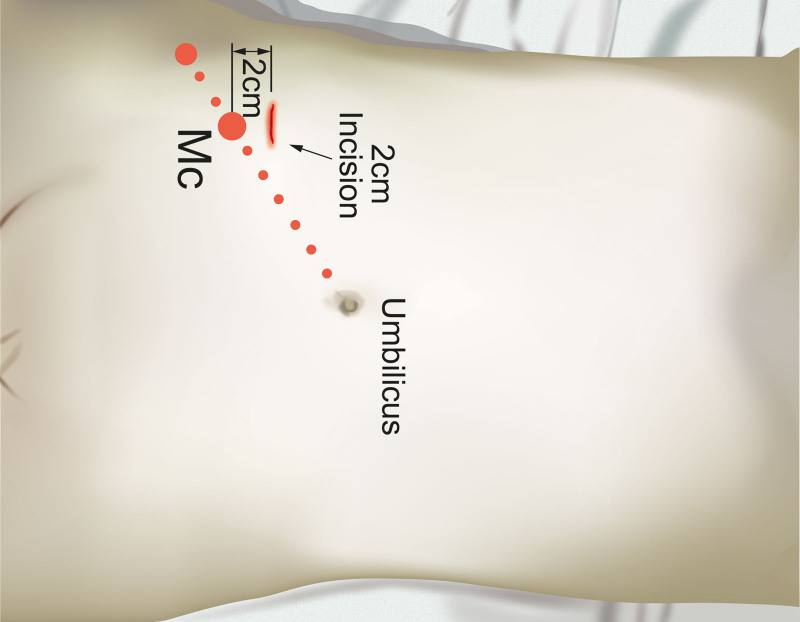
Design of lateral approach incision.

**Figure 7.**
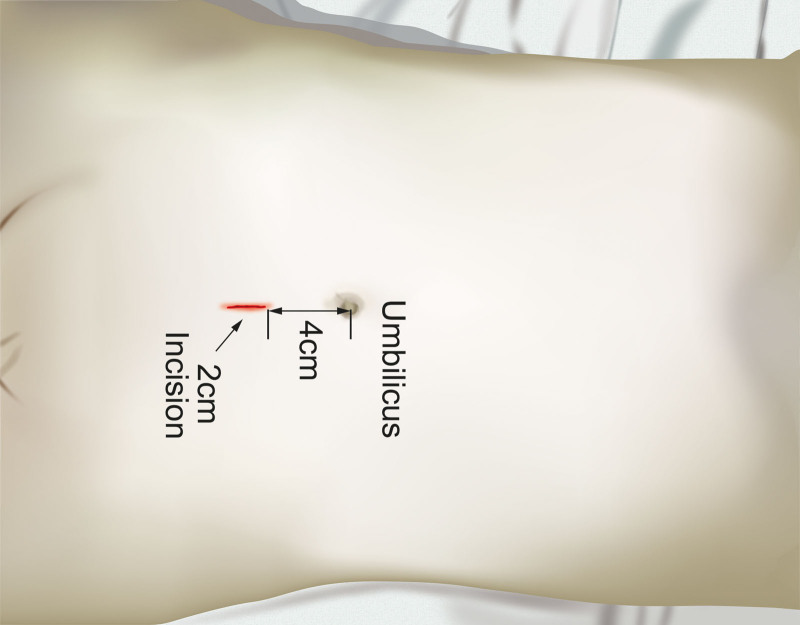
Design of arcuate line incision.

SILS for laparoscopic hernia repair was first performed by Cugura in 2009, and the use of a single laparoscopic port through an umbilical incision resulted in a scar-free procedure.^[47]^ Single-port LIHR can be performed in 2 ways. One is the placement of a multichannel trocar through a single hole; the other is the placement of multiple functionally independent trocars through the umbilicus. The single-port TAPP procedure is similar to the conventional TAPP procedure. Single-port TEP repair has progressed more rapidly, and many novel ideas have been proposed, including the posterior sheath approach, lateral approach, and Arcuate approach. The posterior approach to the posterior sheath establishes an operating space at the umbilical border and enters the posterior layer of the posterior sheath (Fig. 5). There are several advantages to the posterior sheath approach: Incision design: The median incision has certain advantages in the treatment of bilateral hernias. Port placement: The base is fully deployed, stabilizing the port and facilitating smoother operations. Impact of peritoneal rupture on surgical procedures: After the peritoneum is damaged through the posterior sheath approach, vertical movement of the peritoneum may affect the operation, but the peritoneum itself is soft and has little impact on the operation; additionally, the creeping phenomenon is rare. Configuration: There is no contralateral restriction, which creates a larger preperitoneal space.^[48,49]^ The lateral approach has certain advantages for the treatment of complex hernias (Fig. 6); it provides not only a new scheme for patients with contraindications or relative contraindications to midline port placement but also a new perspective for observing the membrane anatomy in the inguinal region from the outside to the inside. However, there are also the following inherent shortcomings: it is limited to the management of unilateral hernias and can be relatively difficult to apply for the management of contralateral hernias; and the skin incision cannot be hidden, resulting in a poor cosmetic effect.^[50]^ In the bowline approach, the incision is moved down to 4 cm below the umbilicus (Fig. 7) such that the bowline is located in the center of the incision, which provides several advantages: the “chopstick” effect on the instruments is significantly reduced, and various fine operations are easier; less space is needed for separation, causing less injury and less postoperative pain; better protection of the blood vessels and ureters is achieved; and the same incision can be used for the management of bilateral inguinal hernias.^[51]^ SILS conceals the surgical incision in the umbilicus or at the umbilical margin, resulting in less pain, faster recovery, no significant difference in complications, and good cosmetic results; this method is safe and effective compared with conventional three-port laparoscopic hernia repair, with the disadvantage that SIL-TAPP repair may require a longer operation, as the procedure is more difficult and has a steeper learning curve.^[52,53]^ SILS-TEP repair is also applicable to patients with a previous history of open hernia repair and elderly patients.^[54–56]^

### 3.4. Robotic inguinal hernia repair: the future of LIHR

Since the first robot-assisted laparoscopic inguinal hernioplasty was carried out in the United States in 2007, the number of robot-assisted inguinal hernioplasties worldwide has increased every year.^[57]^ Robotic inguinal hernia repair is a natural step in the progression of LIHR that is based on the same surgical principles but involves key advances. For doctors, robotic surgery can provide a three-dimensional view of the surgical field, with high magnification of the surgical field, a clearer view of the anatomy and less tissue damage. At the same time, the instruments feature a wrist joint, which greatly improves the ergonomic experience of the traditional “straight stick” instruments used in laparoscopic surgery. This joint provides great flexibility, especially in upper peritoneal dissociation, which has obvious advantages; the upper peritoneal region can be dissociated to a greater extent in the upper and forward directions,^[58]^ and the advantages of continuous suturing when closing the peritoneum are more obvious under robotic assistance.^[59]^ For patients, robotic inguinal hernia repair offers the benefit of a minimally invasive approach without the need for any penetrating fixation (staples) methods and the benefit of allowing minimally invasive surgical access in very challenging situations. As with laparoscopic surgery, both robotic total extraperitoneal (rTEP) and robotic transabdominal preperitoneal (rTAPP) repair are feasible; however, because of technical factors, rTAPP repair is performed much more frequently than rTEP repair.^[44,60]^ Compared with traditional laparoscopic approaches, robotic approaches have similar early clinical outcomes in terms of postoperative pain, quality of life, mobility, cosmesis, wound-related morbidity and complications. However, robotic approaches are more costly and require longer operations.^[61,62]^ Robotic inguinal hernia repair is currently performed at only a few hospitals, where it is available(Table 1).

**Table 1:** Summarize various methods in terms of concept, advantages, disadvantages and development.

Operation	Concept	Advantage	Disadvantage	Development
IPOM	Intraabdominal peritoneal onlay mesh repair	1. Simple and easy to learn	1. Significant postoperative pain 2. Severe abdominal adhesions 3. The recurrence rate is high	Applied to 1. Incision hernia 2. Recurrent hernia
TAPP	Transabdominal laparoscopic preperitoneal hernia repair	1. Simpler than TEP 2. Wide surgical field of vision 3. Recognizable anatomical structure	1. Easy to be disturbed by abdominal organs 2. Prone to abdominal adhesions	Applied to 1. Large hernial sac 2. Incarcerated hernia 3. Female patients
TEP	Totally Extraperitoneal hernia repair	1. Avoiding interference from abdominal organs 2. Compared with TAPP, the surgery has less damage, shorter surgery time, and less postoperative pain	1. High operational difficulty 2. Small surgical field of view 3. Difficult to identify anatomical structures	Applied to 1. Straight hernias, 2. Bilateral hernias 3. Elderly patients
SILS	Single port laparoscopic Inguinal hernia repair	1. Mild pain 2. Quicker recovery 3. Good cosmetic effect	1. Long surgical time 2. High surgical difficulty	Different approaches have emerged, each with its own advantages
Robotic inguinal hernia repair	Robotic inguinal hernia repair	1. Greater surgical field of view 2. Clearer dissection 3. Reduced tissue damage	1. Longer surgical time 2. High cost	Robotic inguinal hernia repair is currently performed at only a few hospitals, where it is available

IPOM = intraabdominal peritoneal onlay mesh repair, SILS = single-port laparoscopic technique, TAPP = transabdominal laparoscopic preperitoneal hernia repair, TEP = totally extraperitoneal hernia repair.

## 4. Mesh: an indispensable and important component of LIHR

In the 1960s, the new therapeutic concept of patching, which involved the deployment of a flat mesh over the hernial defect to strengthen the groin, began to spread. In 1984, Lichtenstein, an American surgeon, used a patch made of polypropylene for surgical hernia repair, after which artificial materials were widely used in hernia and abdominal wall surgery.^[63]^ In the beginning, synthetic patches were used, which were divided into absorbable and nonabsorbable patches; absorbable patch materials included polygelatin ester, polyhydroxyacetic acid, and polypolysaccharide 910; nonabsorbable patch materials included polypropylene, polytetrafluoroethylene, and polyethylene terephthalate.^[64]^ Additionally, biological patches made of decellularized and autologous tissues and composite patches, such as Bard composite patches, have been used. Patches have also been modified in terms of texture, with the application of both standard flat patches and 3D patches; additionally, patch usage has increasingly shifted from heavy to light.^[65]^ Considering the special physiological and morphological features of the groin and the specificity of the source of degenerative inguinal hernias, the traditional treatment model based on a simple static flat mesh over the defect no longer seems to meet the need to address the multiple aspects of the disease. Instead, the ideal therapeutic model should utilize tools capable of handling the natural dynamic cyclic loading of the inguinal muscles in a more physiological manner. This new therapeutic model should also be based on an innovative device that, unlike the low-quality biological response of the platysmal mesh, should induce completely consistent regeneration of the structures that make up the inguinal barrier. One possible theory is to use the same polypropylene material as in the conventional implant but modify the contour into a 3D shape with inherent elasticity and intrinsic memory. The hypothesis underlying these attempts includes the design of a 3D structure capable of imparting the desired dynamic response that translates the biological response into a regenerative effect rather than the common foreign body response to traditional hernia implants. ProFlor is a specially designed multilayered 3D scaffold with reinforced edges. ProFlor has a proprietary dynamic response capability and can be introduced into the hernia opening for permanent closure. Due to its inherent centrifugal expansion, when placed, the ProFlor scaffold completely repairs the hernia without the need for fixation. After years of research, it is now certain that ProFlor ushers in a new era in the treatment of inguinal hernias. It is a new therapeutic modality for the treatment of inguinal hernias that is fully compatible with inguinal physiology and is ideal for overcoming degenerative injuries caused by inguinal hernias. Four new, advanced concepts for the treatment of inguinal hernias are embodied in this new hernia fixator: regenerative stenting, dynamic responsive behavior, permanent defect occlusion, and freedom from fixation. Due to these innovative features, ProFlor may represent a turning point in the treatment of inguinal hernias.^[66]^ Patch fixation methods include bonding with medical adhesive, stapling with a stapler and placing absorbable sutures. At present, there is no consensus on the best method for mesh fixation. Based on evaluations of the existing methods, stapling is the fastest fixation method. In terms of cost, medical adhesives and absorbable sutures are relatively inexpensive and affordable. The reported postoperative pain index was the lowest for medical adhesives, followed by absorbable sutures, and then by staples. Each patch fixation method has its own advantages and limitations. No matter what fixation method is used, the goal is to fully unfold the patch after relieving the pneumoperitoneum pressure during the operation, without curling or warping of the edge, so that the patch can become peritoneum as soon as possible. At the same time, patch fixation can reduce the postoperative pain of patients, prevent complications, and reduce medical costs, thus improving the satisfaction of patients and their families.^[67–69]^ However, the need for fixation of the patch is also controversial, with proponents of fixation arguing that displacement, rolling, or crumpling of the patch can lead to hernia recurrence. Scholars who advocate against fixation argue that fixation is not necessary as long as the patch is sufficiently large because adequate separation of the anterior peritoneal space and the use of a sufficiently large patch are more useful in preventing recurrence than fixation.

## 5. LIHR in different populations

**Figure 8.**
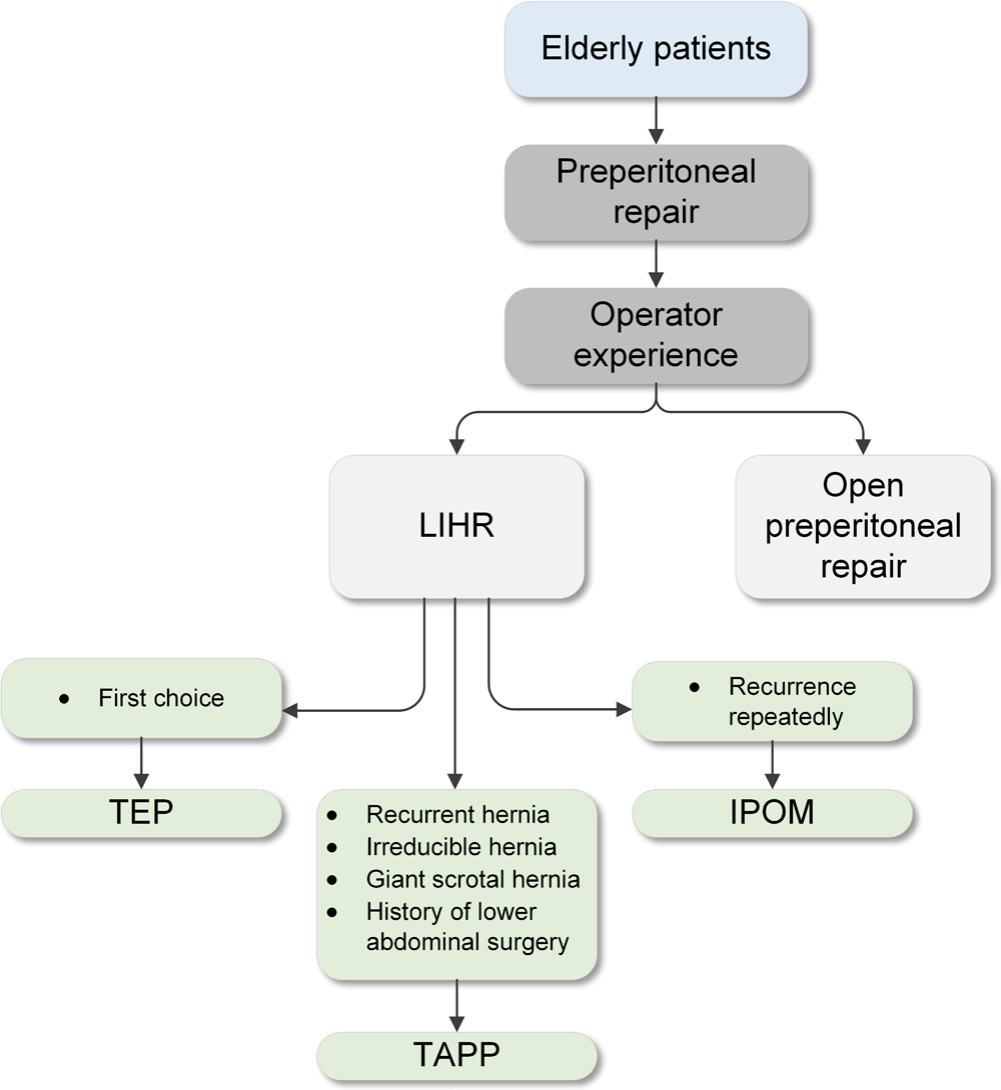
Flow chart for surgical selection of elderly patients. IPOM = intraabdominal peritoneal onlay mesh repair, LIHR = laparoscopic inguinal hernia repair, TAPP = transabdominal laparoscopic preperitoneal hernia repair, TEP = totally extraperitoneal hernia repair.

Older adults: LIHR is safe for older adults. An analysis of 24,571 patients from the Herniamed Registry showed an increase in perioperative complications and reoperation after LIHR that was not only affected by age but also by other factors, such as bilateral surgery, large or scrotal hernia, and higher American Society of Anesthesiologists (ASA) classification, as well as a multitude of risk factors. It has also been shown that the incidence of postoperative complications increases with age from the age of 80 years. Therefore, age > 65 years does not constitute a risk factor for LIHR. In elderly individuals, TAPP repair carries an increased risk of postoperative and general complications and is associated with a longer operation and hospital stay.^[70]^ However, in addition to being safe in elderly individuals, TEP repair can be safely performed under local anesthesia if desired, as demonstrated by Frezza and Ferzli.^[71]^ It should be emphasized that with the diversity of surgical techniques, the management of inguinal hernias will ultimately be based on surgeon expertise, patient- and hernia-related factors, available resources, and logistics. Therefore, there is no single correct approach for the management of inguinal hernias in the general surgical population, let alone elderly individuals; nevertheless, but we believe that certain guidelines should be followed.^[72]^ In patients ≥ 65 years old but ≤ 80 years old, the treatment method is usually selected according to the flow chart for surgical method selection (Fig. 8).^[73]^ TEP repair is the first choice. In people over 80 years old, the criteria for choosing TEP repair are stricter. In addition to the hernia classification, “extraperitoneal” CO_2_ pneumoperitoneum is also a factor to be considered. The preperitoneal space of elderly patients is relatively loose. With extension of the operation time in TEP repair, CO_2_ may be absorbed by tissues, causing an increase in PaCO_2_, subcutaneous emphysema and other pathophysiological changes. This change has little effect under general anesthesia because CO_2_ can be rapidly discharged from the body through mechanical ventilation at the end of the operation, allowing various pathophysiological indicators to return to normal.^[74]^ Therefore, for patients over 80 years old, there are several suggestions for reference. First, perform LIHR under general anesthesia. Second, predict the operation time. For some complex hernias, it is expected that the operation will be longer; thus, TAPP repair can be selected. Third, keep the operation as short as possible. Teenagers: At present, the most commonly used treatment for adolescent inguinal hernia is laparoscopic percutaneous extraperitoneal hernial sac high ligation. Different surgical methods can be used for teenagers of different ages. Laparoscopic high ligation of the hernial sac combined with covering of the medial umbilical fold flap can be used to strengthen the internal ring mouth in patients 10 to 14 years of age. A peritoneal flap can be made from the medial umbilical fold to cover and strengthen the internal ring mouth in patients 14 to 19 years of age.^[75]^ Additionally, biological patches have the advantages of promoting tissue regeneration and degrading over time without causing increased chronic pain, local foreign body sensations or other complications and thus provide a new direction for the treatment of adolescent inguinal hernia. Women: Hernia management is not recommended in women during pregnancy, and the mode of delivery should not be changed. Females with femoral hernias can be treated with LIHR as the first choice.^[38,76]^ Transverse ligament transection in female LIHR will not increase the incidence of pain during sexual intercourse, dysmenorrhea, chronic pelvic pain, uterine prolapse, postoperative seroma or postoperative recurrence and has the advantage of a relatively short operation time.^[77–79]^ However, in patients with fertility requirements, it is recommended to retain the round ligament of the uterus as much as possible.^[80]^

## 6. Conclusion and future perspectives

The rapid development of medical technology and increased understanding of the anatomy of the inguinal region have promoted the emergence and improvement of various surgical treatments for inguinal hernia. In addition, as minimally invasive methods are becoming more common, TAPP and TEP surgical methods become more standardized, the choice of single-port laparoscopic hernioplasty approach, the advantages of robotic inguinal hernioplasty and the application of new patches will become the focus of future laparoscopic Inguinal hernia research. In addition, for patients of different ages and sexes, the choice of LIHR and anesthesia will become more personalized. Laparoscopic Inguinal hernia repair is becoming more and more mature and perfect, providing a better choice for surgical treatment of Inguinal hernia. Therefore, the development prospect of inguinal hernia surgery is very optimistic. I believe LIHR will have a better future.

## Author contributions

**Writing – original draft:** Li shuai xu, Qian Li, Ye Wang, Jiawei Wang, Chengwei Wu, Song Wang, Tingting Cao.

**Writing – review & editing:** Li Xu, Xiaoxu Huang, Yabin Xia.
